# 2,4-Dichloro-*N*-(1,3-thia­zol-2-yl)benzamide

**DOI:** 10.1107/S1600536810044193

**Published:** 2010-11-06

**Authors:** Sohail Saeed, Naghmana Rashid, Wing-Tak Wong

**Affiliations:** a Department of Chemistry, Research Complex, Allama Iqbal Open University, Islamabad, Pakistan; b Department of Chemistry, University of Hong Kong, Pokfulam Road, Pokfulam, Hong Kong SAR, People’s Republic of China

## Abstract

In the mol­ecular structure of the title compound, C_10_H_6_Cl_2_N_2_OS, the dihedral angle between the benzene plane and the plane defined by the amide functionality is 8.6 (1)°, while the thia­zole ring plane is twisted with respect to the amide plane by 68.71 (5)°. In the crystal, pairs of inter­molecular N—H⋯N hydrogen-bond inter­actions connect the mol­ecules into inversion dimers. π–π inter­actions are also observed between neighbouring thia­zole and phen­yl rings [centroid–centroid distance = 3.5905 (13) Å] and a weak C—H⋯π interaction also occurs.

## Related literature

For the synthesis of related thia­zole derivatives and their application, see: Raman *et al.* (2000 ▶); Yunus *et al.* (2007 ▶, 2008 ▶). For microwave-assisted synthesis of amides, see Wang *et al.* (2008 ▶).
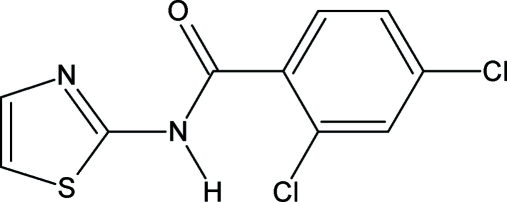


## Experimental

### 

#### Crystal data

C_10_H_6_Cl_2_N_2_OS
*M* *_r_* = 273.13
Monoclinic, 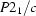

*a* = 14.054 (3) Å
*b* = 13.063 (3) Å
*c* = 6.2880 (14) Å
β = 101.578 (3)°
*V* = 1130.8 (4) Å^3^
*Z* = 4
Mo *K*α radiation
μ = 0.74 mm^−1^
*T* = 304 K
0.38 × 0.27 × 0.07 mm

#### Data collection

Bruker SMART 1000 CCD diffractometer
Absorption correction: multi-scan (*SADABS*; Sheldrick, 1996 ▶) *T*_min_ = 0.768, *T*_max_ = 0.950
5906 measured reflections
1993 independent reflections
1820 reflections with *I* > 2σ(*I*)
*R*_int_ = 0.014

#### Refinement

*R*[*F* ^2^ > 2σ(*F* ^2^)] = 0.029
*wR*(*F* ^2^) = 0.080
*S* = 1.06
1993 reflections
149 parameters
H atoms treated by a mixture of independent and constrained refinement
Δρ_max_ = 0.25 e Å^−3^
Δρ_min_ = −0.24 e Å^−3^

### 

Data collection: *SMART* (Bruker, 1998 ▶); cell refinement: *SAINT* (Bruker, 2006 ▶); data reduction: *SAINT* and *CrystalStructure* (Rigaku/MSC, 2006 ▶); program(s) used to solve structure: *SHELXS97* (Sheldrick, 2008 ▶); program(s) used to refine structure: *SHELXL97* (Sheldrick, 2008 ▶); molecular graphics: *ORTEPII* (Johnson, 1976 ▶) and *Mercury* (Macrae *et al.*, 2008 ▶); software used to prepare material for publication: *SHELX97*.

## Supplementary Material

Crystal structure: contains datablocks global, I. DOI: 10.1107/S1600536810044193/im2236sup1.cif

Structure factors: contains datablocks I. DOI: 10.1107/S1600536810044193/im2236Isup2.hkl

Additional supplementary materials:  crystallographic information; 3D view; checkCIF report

## Figures and Tables

**Table 1:** Hydrogen-bond geometry (Å, °) *Cg*1 is the centroid of the thia­zole ring.

*D*—H⋯*A*	*D*—H	H⋯*A*	*D*⋯*A*	*D*—H⋯*A*
N2—H2*N*⋯N1^i^	0.79 (2)	2.09 (2)	2.880 (2)	178 (2)
C1—H1⋯*Cg*1^ii^	0.93	2.81	3.501 (2)	132

Symmetry codes: (i) 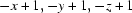; (ii) 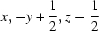.

**Fig. 1.**
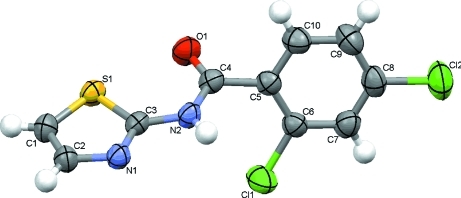
ORTEP plot of the compound with thermal ellipsoids at the 50% probability level and showing the atom numbering scheme.

**Fig. 2.**
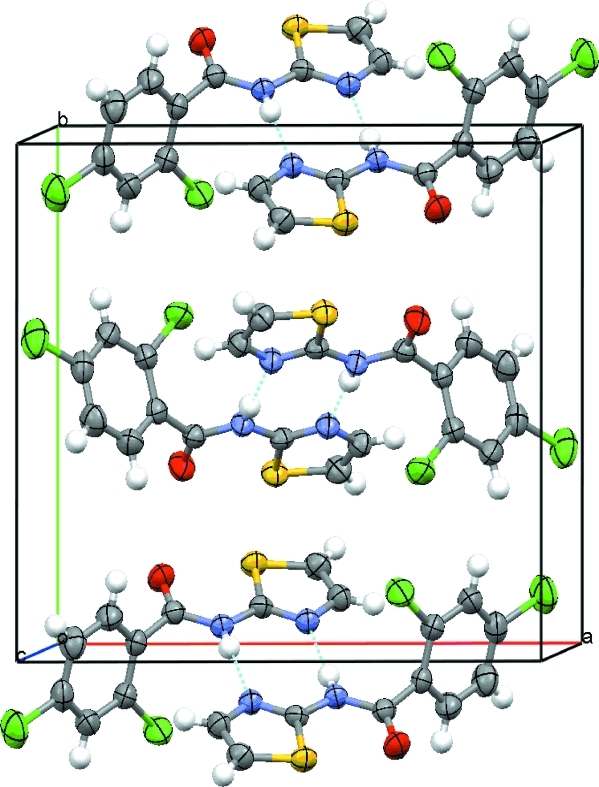
Packing diagram.

## supplementary crystallographic information

### Comment

Substituted and unsubstituted thiazole derivatives are of great importance in biological systems due to their vast range of biological activities such as anti-inflammatory, analgestic and antipyretic (Raman *et al.*, 2000; Yunus *et al.*, 2007, 2008). On the other hand, amide compounds have extensive applications in pharmaceutical industry (Wang *et al.*, 2008).

The title compound, 2,4-dichloro-*N*-thiazol-2-yl-benzamide, C_10_H_6_Cl_2_N_2_OS, crystallizes in the monoclinic space group *P*2_1_/*c* (#14). The molecule is not planar showing a dihedral angle of 8.6 (1)° of the amide group, C3—C5/N2/O1 with respect to the phenyl ring plane, C5—C10/Cl1/Cl2. The thiazolyl ring, C1—C3/N1/S1, is twisted (68.71 (5)°) relative to the amide group. In additon, the phenyl ring plane makes a dihedral angle of 74.89 (5)° with the thiazole ring plane.

There are pair-wise inter-molecular N2—H2N···N1 H-bond interactions linking the molecules into dimeric arrangements. There are also π–π interactions between neighbouring thiazole, S1/N1/C1—C3 (*Cg1*), and phenyl rings, C5—C10 (*Cg2*), in the crystal lattice. The distance between ring centroids *Cg1* and *Cg2* is 3.5905 Å, and dihedral angle between them is determined to 0°.

There is no residual solvent accessible void volume in the unit cell.

### Experimental

A mixture of 2,4-dichlorobenzoyl chloride (0.01 mol) and 2-aminothiazole (0.01 mol) was refluxed in acetone (50 ml) for 1.5 h. After cooling to room temperature, the mixture was poured into acidified cold water. The resulting solid was filtered and washed with cold acetone (yield: 72%). Single crystals of the title compound suitable for single-crystal X-ray analysis were obtained by recrystallization of the light yellow solid from ethyl acetate.

### Refinement

The structure was solved by direct methods (*SHELXS97*) and expanded using Fourier techniques. All non-H atoms were refined anisotropically. C-bound H atoms are all placed geometrically C—H = 0.93 Å for phenyl H-atoms. They were refined using a riding model with *U*_iso_(H) = 1.2 *U*_eq_ (Carrier). N-bound H atoms were located from difference Fourier map and are refined isotropically.

Highest peak is 0.25 at (0.9753, 0.3236, 0.26385) [0.97Å from Cl2] Deepest hole is -0.24 at (0.2315, 0.6265, 0.1208) [0.79Å from Cl1]

### Crystal data

**Table:**

C_10_H_6_Cl_2_N_2_OS	*F*(000) = 552
*M**_r_* = 273.13	*D*_x_ = 1.604 Mg m^−^^3^
Monoclinic, *P*2_1_/*c*	Melting point: 487 K
Hall symbol: -P 2ybc	Mo *K*α radiation, λ = 0.71073 Å
*a* = 14.054 (3) Å	Cell parameters from 6065 reflections
*b* = 13.063 (3) Å	θ = 2.2–25.0°
*c* = 6.2880 (14) Å	µ = 0.74 mm^−^^1^
β = 101.578 (3)°	*T* = 304 K
*V* = 1130.8 (4) Å^3^	Block, colourless
*Z* = 4	0.38 × 0.27 × 0.07 mm

### Data collection

**Table:**

Bruker SMART 1000 CCD diffractometer	1993 independent reflections
Radiation source: fine-focus sealed tube	1820 reflections with *I* > 2σ(*I*)
graphite	*R*_int_ = 0.014
ω scans	θ_max_ = 25.0°, θ_min_ = 2.2°
Absorption correction: multi-scan (*SADABS*; Sheldrick, 1996)	*h* = −16→15
*T*_min_ = 0.768, *T*_max_ = 0.950	*k* = −15→13
5906 measured reflections	*l* = −6→7

### Refinement

**Table:**

Refinement on *F*^2^	Primary atom site location: structure-invariant direct methods
Least-squares matrix: full	Secondary atom site location: difference Fourier map
*R*[*F*^2^ > 2σ(*F*^2^)] = 0.029	Hydrogen site location: inferred from neighbouring sites
*wR*(*F*^2^) = 0.080	H atoms treated by a mixture of independent and constrained refinement
*S* = 1.06	*w* = 1/[σ^2^(*F*_o_^2^) + (0.0375*P*)^2^ + 0.4293*P*] where *P* = (*F*_o_^2^ + 2*F*_c_^2^)/3
1993 reflections	(Δ/σ)_max_ < 0.001
149 parameters	Δρ_max_ = 0.25 e Å^−^^3^
0 restraints	Δρ_min_ = −0.24 e Å^−^^3^

### Special details

**Table:**

Geometry. All e.s.d.'s (except the e.s.d. in the dihedral angle between two l.s. planes) are estimated using the full covariance matrix. The cell e.s.d.'s are taken into account individually in the estimation of e.s.d.'s in distances, angles and torsion angles; correlations between e.s.d.'s in cell parameters are only used when they are defined by crystal symmetry. An approximate (isotropic) treatment of cell e.s.d.'s is used for estimating e.s.d.'s involving l.s. planes.
Refinement. Refinement of *F*^2^ against ALL reflections. The weighted *R*-factor *wR* and goodness of fit *S* are based on *F*^2^, conventional *R*-factors *R* are based on *F*, with *F* set to zero for negative *F*^2^. The threshold expression of *F*^2^ > σ(*F*^2^) is used only for calculating *R*-factors(gt) *etc*. and is not relevant to the choice of reflections for refinement. *R*-factors based on *F*^2^ are statistically about twice as large as those based on *F*, and *R*- factors based on ALL data will be even larger.

### Fractional atomic coordinates and isotropic or equivalent isotropic displacement parameters (Å^2^)

**Table:**

	*x*	*y*	*z*	*U*_iso_*/*U*_eq_	
Cl1	0.24941 (4)	0.64173 (4)	0.24448 (11)	0.0739 (2)	
Cl2	0.02120 (5)	0.61769 (6)	0.82771 (11)	0.0849 (2)	
S1	0.41434 (3)	0.32850 (4)	0.00755 (7)	0.04892 (15)	
O1	0.25039 (10)	0.34891 (11)	0.1798 (2)	0.0592 (4)	
N1	0.53725 (10)	0.42950 (11)	0.2878 (2)	0.0452 (3)	
N2	0.38052 (10)	0.43647 (12)	0.3593 (3)	0.0441 (3)	
C1	0.53294 (15)	0.33469 (15)	−0.0224 (3)	0.0536 (5)	
H1	0.5568	0.3037	−0.1343	0.064*	
C2	0.58651 (14)	0.39011 (15)	0.1371 (3)	0.0500 (4)	
H2	0.6525	0.4013	0.1457	0.060*	
C3	0.44612 (12)	0.40332 (12)	0.2379 (3)	0.0399 (4)	
C4	0.28500 (12)	0.41126 (13)	0.3182 (3)	0.0436 (4)	
C5	0.22451 (12)	0.46497 (13)	0.4549 (3)	0.0435 (4)	
C6	0.20114 (12)	0.56805 (14)	0.4270 (3)	0.0471 (4)	
C7	0.13838 (13)	0.61528 (15)	0.5405 (3)	0.0549 (5)	
H7	0.1220	0.6839	0.5175	0.066*	
C8	0.10102 (13)	0.55811 (18)	0.6880 (3)	0.0571 (5)	
C9	0.12440 (15)	0.45612 (18)	0.7240 (4)	0.0637 (5)	
H9	0.0997	0.4191	0.8271	0.076*	
C10	0.18494 (14)	0.40987 (16)	0.6047 (3)	0.0561 (5)	
H10	0.1994	0.3407	0.6252	0.067*	
H2N	0.4017 (14)	0.4739 (16)	0.456 (3)	0.048 (5)*	

### Atomic displacement parameters (Å^2^)

**Table:**

	*U*^11^	*U*^22^	*U*^33^	*U*^12^	*U*^13^	*U*^23^
Cl1	0.0751 (4)	0.0560 (3)	0.1005 (5)	0.0071 (3)	0.0411 (3)	0.0226 (3)
Cl2	0.0658 (4)	0.1064 (5)	0.0910 (5)	0.0030 (3)	0.0361 (3)	−0.0242 (4)
S1	0.0525 (3)	0.0473 (3)	0.0460 (3)	−0.00460 (19)	0.0078 (2)	−0.01145 (19)
O1	0.0506 (8)	0.0593 (8)	0.0655 (9)	−0.0091 (6)	0.0061 (6)	−0.0187 (7)
N1	0.0420 (8)	0.0449 (8)	0.0487 (8)	−0.0020 (6)	0.0095 (6)	−0.0084 (6)
N2	0.0398 (8)	0.0430 (8)	0.0485 (8)	−0.0022 (6)	0.0064 (6)	−0.0130 (7)
C1	0.0596 (11)	0.0544 (11)	0.0499 (10)	−0.0013 (9)	0.0186 (9)	−0.0099 (8)
C2	0.0471 (10)	0.0517 (11)	0.0540 (10)	−0.0015 (8)	0.0168 (8)	−0.0067 (8)
C3	0.0446 (9)	0.0331 (8)	0.0411 (9)	0.0008 (7)	0.0065 (7)	−0.0029 (7)
C4	0.0424 (9)	0.0385 (9)	0.0479 (9)	−0.0003 (7)	0.0042 (7)	−0.0012 (7)
C5	0.0344 (8)	0.0457 (9)	0.0482 (9)	−0.0023 (7)	0.0035 (7)	−0.0020 (7)
C6	0.0399 (9)	0.0460 (10)	0.0555 (10)	−0.0028 (7)	0.0098 (8)	−0.0009 (8)
C7	0.0451 (10)	0.0489 (11)	0.0705 (13)	0.0011 (8)	0.0108 (9)	−0.0080 (9)
C8	0.0402 (9)	0.0740 (14)	0.0580 (11)	−0.0009 (9)	0.0123 (8)	−0.0108 (10)
C9	0.0597 (12)	0.0747 (15)	0.0614 (12)	−0.0019 (11)	0.0234 (10)	0.0083 (11)
C10	0.0534 (11)	0.0518 (11)	0.0635 (12)	0.0001 (9)	0.0123 (9)	0.0086 (9)

### Geometric parameters (Å, °)

**Table:**

Cl1—C6	1.7370 (19)	C2—H2	0.9300
Cl2—C8	1.741 (2)	C4—C5	1.499 (2)
S1—C1	1.716 (2)	C5—C10	1.387 (3)
S1—C3	1.7299 (16)	C5—C6	1.389 (3)
O1—C4	1.219 (2)	C6—C7	1.386 (3)
N1—C3	1.302 (2)	C7—C8	1.374 (3)
N1—C2	1.381 (2)	C7—H7	0.9300
N2—C4	1.356 (2)	C8—C9	1.380 (3)
N2—C3	1.379 (2)	C9—C10	1.381 (3)
N2—H2N	0.79 (2)	C9—H9	0.9300
C1—C2	1.339 (3)	C10—H10	0.9300
C1—H1	0.9300		
			
C1—S1—C3	88.40 (9)	C10—C5—C4	119.82 (16)
C3—N1—C2	109.94 (15)	C6—C5—C4	121.89 (16)
C4—N2—C3	124.36 (15)	C7—C6—C5	121.70 (17)
C4—N2—H2N	120.1 (14)	C7—C6—Cl1	117.84 (15)
C3—N2—H2N	115.5 (14)	C5—C6—Cl1	120.46 (14)
C2—C1—S1	110.84 (14)	C8—C7—C6	118.32 (19)
C2—C1—H1	124.6	C8—C7—H7	120.8
S1—C1—H1	124.6	C6—C7—H7	120.8
C1—C2—N1	115.56 (17)	C7—C8—C9	121.65 (19)
C1—C2—H2	122.2	C7—C8—Cl2	118.02 (17)
N1—C2—H2	122.2	C9—C8—Cl2	120.32 (17)
N1—C3—N2	121.23 (15)	C8—C9—C10	119.04 (19)
N1—C3—S1	115.25 (13)	C8—C9—H9	120.5
N2—C3—S1	123.49 (13)	C10—C9—H9	120.5
O1—C4—N2	122.43 (17)	C9—C10—C5	121.08 (19)
O1—C4—C5	122.07 (15)	C9—C10—H10	119.5
N2—C4—C5	115.51 (15)	C5—C10—H10	119.5
C10—C5—C6	118.16 (17)		

### Hydrogen-bond geometry (Å, °)

**Table:**

Cg1 is the centroid of the thiazole ring.

**Table:**

*D*—H···*A*	*D*—H	H···*A*	*D*···*A*	*D*—H···*A*
N2—H2N···N1^i^	0.79 (2)	2.09 (2)	2.880 (2)	178 (2)
C1—H1···Cg1^ii^	0.93	2.81	3.501 (2)	132

Symmetry codes: (i) −*x*+1, −*y*+1, −*z*+1; (ii) *x*, −*y*+1/2, *z*−1/2.
